# Supplementary material for: Reconciling Mining with the Conservation of Cave Biodiversity: A Quantitative Baseline to Help Establish Conservation Priorities
Source: PLoS One. 2016 Dec 20;11(12):e0168348. doi: 10.1371/journal.pone.0168348 (PMC5173368; doi:10.1371/journal.pone.0168348)
Supplement: S1 Dataset — (ZIP) [file pone.0168348.s002.zip › Taxa/Serra Norte/SN_2013/N4WS/Lista N4WS-16.pdf]

CAVIDADE N4WS-0016							
**Classe**	**Ordem**	**Fam/Outros**	**Gên/Outros**	**Espécie**	**S-U**	**U-S**	Zon.
Arachnida	Acari	(Parasitengonina)		sp.1	X		P
Arachnida	Acari	Argasidae	*Ornithodoros*	sp.1	X		E
Arachnida	Acari	Cunaxidae		sp.1	X		E
Arachnida	Amblypygi	Phrynidae	*Heterophrynus*	*longicornis*	1	1	E
Arachnida	Araneae	Araneidae		jovem		X	E
Arachnida	Araneae	Ochyroceratidae	*Speocera*	sp.1	X	X	P
Arachnida	Araneae	Pholcidae	*Mesabolivar*	*eberhard*	X	X	P
Arachnida	Araneae	Theridiosomatidae		jovem		X	E
Arachnida	Opiliones	Sclerosomatidae		jovem		X	E
Arachnida	Palpigradi	Eukoeneniidae	*Allokoenenia*	sp.1	X		P
Arachnida	Pseudoscorpiones	Chernetidae	*Spelaeochernes*	sp.1	X	X	E/P
Arachnida	Pseudoscorpiones	Chthoniidae	*Pseudochthonius*	sp.1		X	E
Arachnida	Schizomida	Hubbardiidae	aff. *Rowlandius*	sp.1		X	P
Diplopoda	Glomeridesmida	Glomeridesmidae		sp.1		1	E
Diplopoda	Polydesmida	Pyrgodesmidae		sp.1	X		P
Entognatha	Collembola	Paronellidae		sp.1	X		E
Entognatha	Collembola	Paronellidae		sp.4	X	X	P
Insecta	Coleoptera	Tenebrionidae		jovem		X	E
Insecta	Dermaptera	Pygidicranidae		sp.1		1	E
Insecta	Diptera	Cecidomyiidae	Cecidomyiinae	sp.	X		E
Insecta	Diptera	Psychodidae	*Sciopemyia*	*sordellii*		X	E
Insecta	Diptera	Tipulidae	Tipulinae	sp.	X		E
Insecta	Heteroptera	Cydnidae	*Pangaeus*	sp.1	X		E
Insecta	Heteroptera	Reduviidae	Emesinae	sp.1		1	E
Insecta	Heteroptera	Reduviidae	Reduviinae	jovem	1	3	E
Insecta	Homoptera	Cixiidae		jovem		X	P
Insecta	Hymenoptera	Formicidae	*Camponotus*	sp.1	X		E
Insecta	Hymenoptera	Formicidae	*Dolichoderus*	aff. *bispinosus*	X		E/P
Insecta	Hymenoptera	Formicidae	*Octostruma*	sp.1		X	E
Insecta	Isoptera	Termitidae	*Cortaritermes*	*silvestrii*	X		E
Insecta	Lepidoptera			jovem	X		P
Insecta	Neuroptera	Myrmeleontidae		jovem		2	E
Insecta	Orthoptera	Phalangopsidae	*Phalangopsis*	sp.	74	35	P
Insecta	Psocoptera	Psocomorpha		jovem		X	E
Insecta	Psocoptera	Psyllipsocidae	*Psyllipsocus*	sp.2	X	X	E
Insecta	Thysanoptera	Thripidae		sp.1		X	E
Malacostraca	Isopoda	Philosciidae		sp.1	X		P
Malacostraca	Isopoda	Philosciidae		sp.2		X	P
Amphibia	Anura	Strabomantidae	*Pristimantis*	cf. *fenestratus*	1	1	E
Mammalia	Chiroptera			sp.	6		E/P
